# Establishment of a finite element model and stress analysis of intra-articular impacted fragments in posterior malleolar fractures

**DOI:** 10.1186/s13018-022-03043-2

**Published:** 2022-03-28

**Authors:** Wenyong Xie, Hao Lu, Sizheng Zhan, Yijun Liu, Yuan Quan, Hailin Xu, Zhongguo Fu, Dianying Zhang

**Affiliations:** 1 Department of Orthopedics and Trauma, Peking University People’s Hospital, Beijing, 100044 China; 2 Department of Foot and Ankle Surgery, Center for Orthopaedic Surgery, The Third Affiliated Hospital of Southern Medical University, Guangzhou, 510630 China

**Keywords:** Finite element analysis, Ankle fractures, Posterior malleolus, Intra-articular fragment, Post-traumatic arthritis

## Abstract

**Background:**

Intra-articular impacted fragments (IAIFs) are considered articular surface fragments resulting from impact and compressive forces. The malreduction of IAIFs in posterior malleolar fractures has been associated with talar subluxation and long-term post-traumatic arthritis. In this study, we establish IAIF defect finite element models of different sizes in posterior malleolar fractures and explored how IAIF defects predict the onset of post-traumatic arthritis.

**Methods:**

A reliable three-dimensional finite element model of the normal ankle was established. Finite element models with different sizes of IAIF defects were created to calculate ankle joint contact stress. The finite element data were recorded and analyzed.

**Results:**

There was a linear relationship between the size of the IAIF defect and MCS with IAIF defects in the posterolateral region. The result of Pearson linear correlation analysis was *r* = 0.963, *P* = 0.009. The regression equation was MCS = 0.087*AI + 2.951 (AI, area of IAIF) by simple linear regression analysis. When the IAIF defect was in the posteromedial region, there was also a linear relationship between the size of the IAIF defect and MCS. The result of Pearson linear correlation analysis was *r* = 908, *P* = 0.033. The regression equation was MCS = 0.065*AI + 1.841. The MCS was increased mainly in the border of the IAIF defect.

**Conclusions:**

A small IAIF defect in the posterior malleolus will result in a high MCS, and the MCS in the posterolateral region is larger than the MCS in the posteromedial region when the size of the IAIF defect is the same. We obtain the regression equation of MCS and area of IAIF defect. This indicates that patients are more prone to post-trauma arthritis when the size of IAIF defects is more than 17.8 mm^2^ in the posterolateral region and more than 40.9 mm^2^ in the posteromedial region.

*Trial registration* Retrospectively registered.

## Background

The incidence of ankle fractures has been reported to be 10% among all fractures [1]. A system review reported that about 95% ankle fracture were acute fractures [2]. Posterior malleolar fractures can have a high incidence among ankle fractures [3]. It was unstable fractures when ankle fractures involved posterior malleolar fractures and common on supination-external rotation ankle fractures, which were also associated with negative outcome and development of radiographic osteoarthritis [4]. At present, due to the use of CT scans, intra-articular impacted fragments (IAIFs) in ankle fractures are commonly found, and the morphology of IAIFs varies [5–7]. Scheck et al. [8] first described the die-punch fragment, the intra-articular fragment, in distal radius fractures, and subsequently, the intra-articular fracture of the distal tibia was studied [9]. However, only three studies have reported the relevant description of IAIF [5–7].

IAIFs are articular surface fragments that are impacted by the talus. Talus subluxation can be found in ankle fractures with IAIF [5]. Long-term post-traumatic arthritis is related to malreduction of IAIFs [7, 10, 11] and is more likely to occur [12, 13]. Important pathomechanical determinants of post-traumatic arthritis may exist as peak instantaneous contact stresses [14]. Anderson et al. [15] proposed that elevated contact stress exposure would predict the onset of post-traumatic arthritis based on FEA modeling and clinical follow-up. It is considered to be critically important to reduce displaced articular fractures anatomically, which could minimize the risk of developing post-traumatic arthritis [16].

The finite element analysis (FEA) technology had become a common form of biomechanical simulation, which was based on modern computational method and structural mechanics analysis and had many advantages compared to cadaver specimens. In some studies, the technology of FEA was also used in tumor bone and thermal necrosis with many advantages [17, 18]. It was difficult to study the thermal necrosis from drilling bone though cadaver specimens, while the FEA could be well applied [17]. We have already established an IAIF defect finite element model in posterior malleolar fractures and discussed the relation between IAIF defects and post-traumatic arthritis [19].

However, the size of IAIF defects and post-traumatic arthritis, which is of great importance for clinical treatment, has not been studied. Due to equipment issues, costs and ethical issues, FEA technology, which is based on modern computational methods and structural mechanics analysis, is a better choice to study the relationship of different sizes of IAIF defects and post-traumatic arthritis. This would enable the result of FEA to be as realistic in terms of ontology as possible [20].

The aim of this study was to establish IAIF defect finite element models of different sizes in posterior malleolar fractures and explore the IAIF defects and how to predict the onset of post-traumatic arthritis.

## Materials and methods

First, DICOM data from a normal ankle joint CT in our institution were obtained. Then, a finite element model of the ankle joint was established through relevant software. After verifying the stability and reliability of the finite element model, IAIF defects were established in the finite element model. The detailed methods of the above have been published in the journal of Injury [19].

**Fig. 1:**
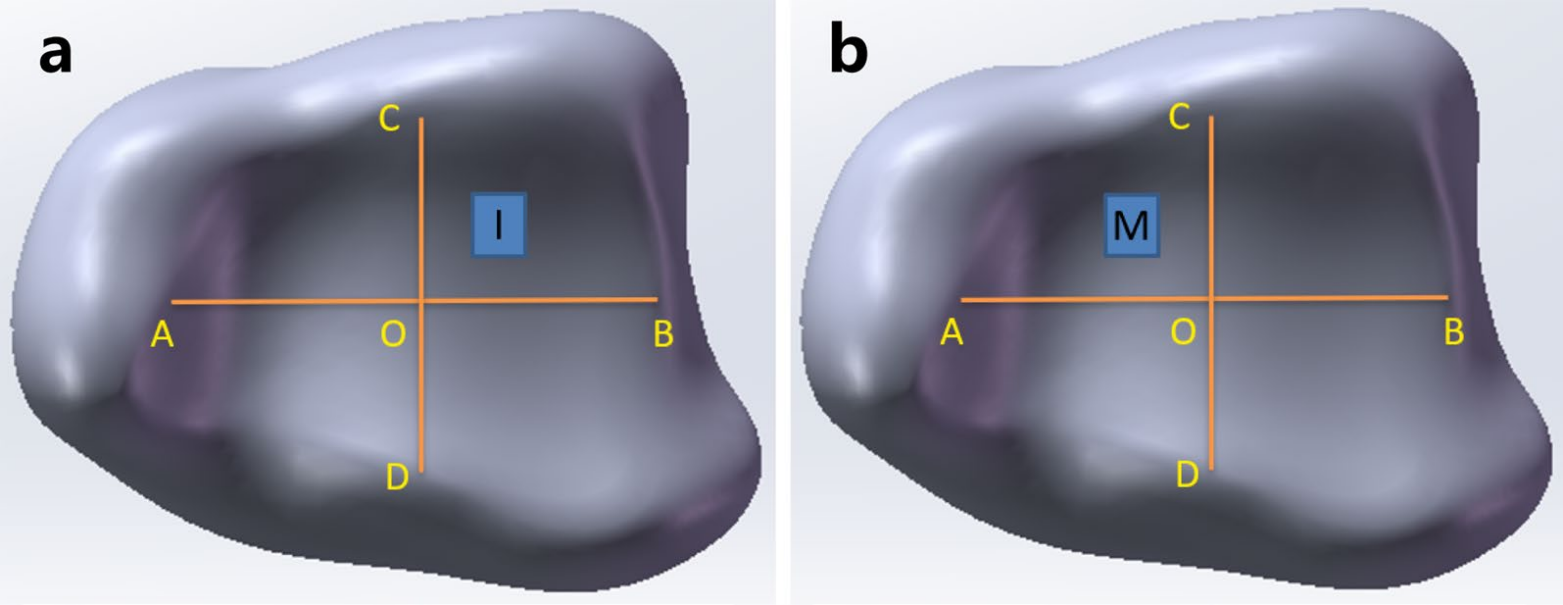
Establishment of IAIF defect. AB line was the midpoint of lateral malleolus to midpoint of medial malleolus and CD line was midpoint of posterior edge of the tibia to midpoint of the anterior edge of the tibia. O point was the intersection point of the AB and CD line. **a** I fragment was in the center of BOC region and **b** M fragment was in the center of AOC region

The bottom view of the distal tibial articular surface is shown in Fig. 1. The AB line was the midpoint of the lateral malleolus to the midpoint of the medial malleolus, and the CD line was the midpoint of the posterior edge of the tibia to the midpoint of the anterior edge of the tibia. The O point was the intersection point of the AB and CD lines, so the posterior malleolus was divided into region AOC (posteromedial region) and region BOC (posterolateral region). Almost all IAIF presented at the posterolateral and posteromedial regions of the posterior malleolus [5–7]. We established IAIF defects in the cartilage and subchondral bone in the center of the posterolateral region (fragment I) and posteromedial region (fragment M). There was no description of the relevant IAIF defect in the existing literature. From the description of IAIF in some studies [5–7], we started a defect IAIF depth with cartilage and subchondral bone missing at 2 mm, area with a size of 1 × 2, 3 × 4, 4 × 5.5, 5 × 6.4 and 6 × 7 mm^2^ (Fig. 1).

Statistical analysis was done using SPSS 23.0. A *P* value < 0.05 was considered significant. The relation between the size of the IAIF defect and maximum contact stress was analyzed with Pearson correlation analysis. Simple linear regression analysis was carried out to obtain a regression equation when there was a linear correlation between the size of the IAIF defect and the maximum contact stress.

## Results

**Fig. 2:**
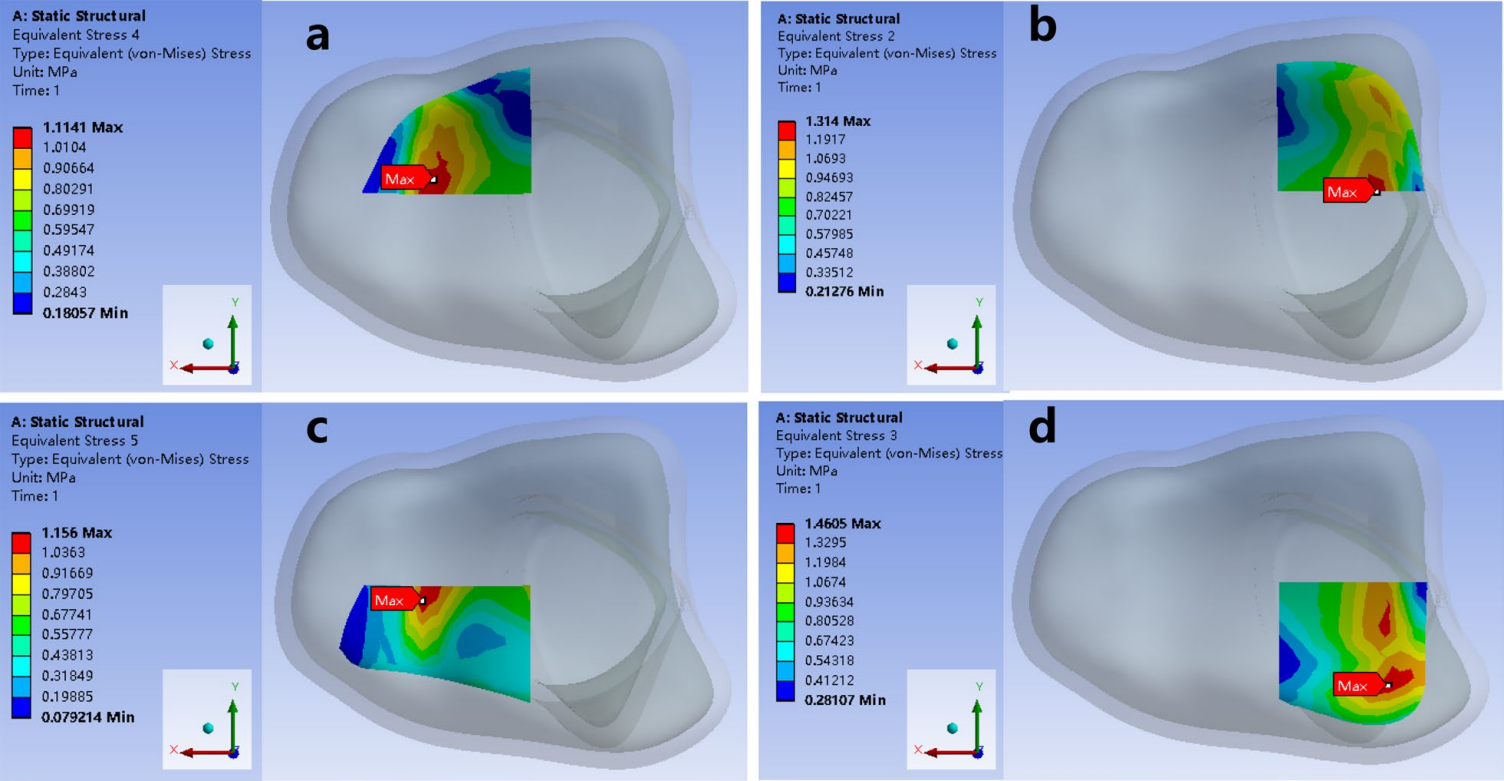
Contact stress distribution of normal ankle joint in four quadrants of region AOC, BOC, AOD and BOD

Finite element analysis of different sizes of IAIF defects: We recorded the maximum contact stress in different sizes of IAIF defects in the posterolateral region or posteromedial region. We also recorded the maximum contact stress in four quadrants of regions AOC, BOC, AOD and BOD (Fig. 2, Table 1).

**Table 1:** Maximum contact stress on the distal tibial articular surface in the different size of intra-articular impacted fragment (IAIF) defect

Size of defect	Maximum contact stress (IAIF Defect in BOC, MPa)					Maximum contact stress (IAIF Defect in AOC, MPa)				
(mm^3^)	Intact	BOC	AOC	BOD	AOD	Intact	BOC	AOC	BOD	AOD
1 × 2 × 2	3.3465	3.3465	1.1558	1.4459	1.1432	1.4425	1.2966	1.4137	1.4425	1.1432
3 × 4 × 2	3.9653	3.9653	1.1757	1.4588	1.148	3.2958	1.303	3.2958	1.4473	1.1612
4 × 5.5 × 2	4.7455	4.7455	1.1639	1.4732	1.15	3.49	1.3022	3.49	1.4496	1.1974
5 × 6.4 × 2	5.1854	5.1854	1.1843	1.4935	1.1679	3.5719	1.307	3.5719	1.4496	1.2457
6 × 7 × 2	7.089	7.089	1.2162	6.5087	5.4433	4.5605	1.3185	4.5605	1.5378	1.2806

*IAIF* intra-articular impacted fragment

**Fig. 3:**
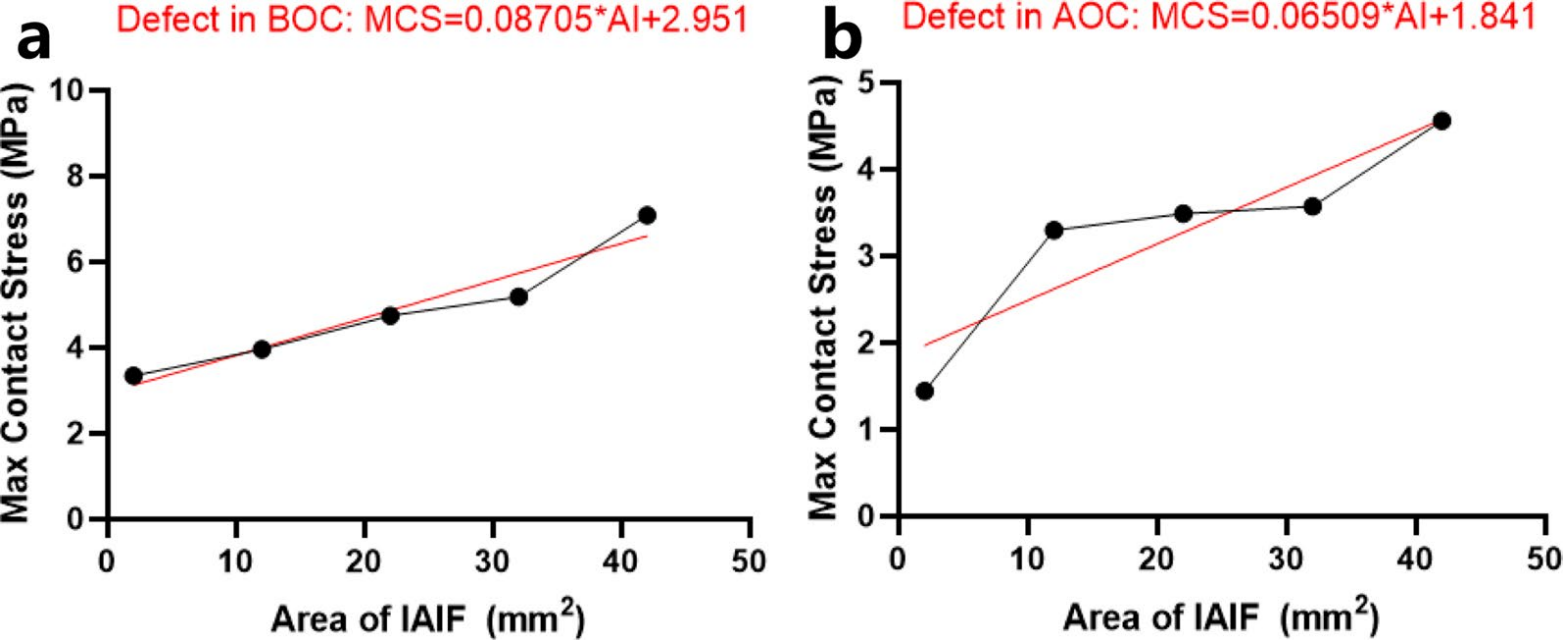
Relation and simple linear regression between MCS (maximum contact stress) and AI (area of IAIF) though GraphPad Prism: **a** MCS = 0.087*AI + 2.951, *P* = 0.009, **b** MCS = 0.065*AI + 1.841, *P* = 0.033

The data of the size of the IAIF defect and maximum contact stress (MCS) were input into SPSS 23.0 to analyze their correlation. The *X* axis is taken as the size of the IAIF defect, and the *Y* axis is taken as the MCS. When the IAIF defect was in the posterolateral region, there was a linear relationship between the size of the IAIF defect and MCS. The results of Pearson linear correlation analysis were *r* = 0.963, *P* = 0.009. The MCS was positively correlated with the size of the IAIF defect. The regression equation was MCS = 0.087*AI + 2.951 (AI, area of IAIF) by simple linear regression analysis. When the IAIF defect was in the posteromedial region, there was also a linear relationship between the size of the IAIF defect and MCS. The results of Pearson linear correlation analysis were *r* = 908, *P* < 0.033. It was positively correlated between the size of the IAIF defect and MCS. The regression equation was MCS = 0.065*AI + 1.841, although simple linear regression analysis was performed (Fig. 3).

## Discussion

Through finite element analysis, it is found that a small IAIF defect in the posterior malleolus will result in a high MCS, and the MCS in the posterolateral region is larger than the MCS in the posteromedial region when the size of the IAIF defect is the same. There is an obvious linear correlation between the size of the IAIF defect and the MCS. Therefore, the MCS of any IAIF can be calculated and then further discuss the relation between IAIF defects and traumatic arthritis. We established IAIF defect finite element models with sizes of 1 × 2, 3 × 4, 4 × 5.5, 5 × 6.4 and 6 × 7 mm^2^ in the posterolateral region and posteromedial region. The relation between IAIF defects and MCS has not been studied before. The characteristics of IAIF make it easy to ignore in surgery, which can be vulnerable to malreduction or loss.

The intact anatomical structure plays an important role in the stability and function of the ankle joint. Chronically elevated contact stresses resulting from articular incongruity overload the cartilage and may be important pathomechanical determinants of post-traumatic arthritis [14, 21]. A high occurrence of IAIF in posterior malleolar fractures is considered the major driver of articular incongruity leading to post-traumatic arthritis. Sultan et al. [7] reported that IAIF was found in 43% of posterior malleolar fractures, and the position of IAIF was posterolateral in 64%, midposterior in 19% and posteromedial in 17% of cases. IAIF has not received enough attention during the operation, and it was reported that more than 40% of cases of posterior malleolar fractures with IAIF were considered poor reduction [7].

Our research results showed that when the IAIF defect was in the posterolateral region, a small defect of 2 mm^2^ made the maximum contact stress more than double that of the normal ankle joint. When the IAIF defect increased, the maximum contact stress increased gradually. There was a sharp increase in defect of 32–42 mm^2^. We obtained a regression equation of MCS = 0.087*AI + 2.951. Therefore, we could know the relative MCS of different sizes of IAIF defects. There were insignificant changes when the IAIF defect was in the posteromedial region with a defect of 2 mm^2^. The greatest increase in MCS occurred in a defect of 2–12 mm^2^, while the defect of 12–22 mm^2^ increased slowly and then had a sharp increase in defects of 32–42 mm^2^. The regression equation was MCS = 0.065*AI + 1.841. From our results, we found that there was a greater influence on MCS with a defect of 32–42 mm^2^ in both the posterolateral and posteromedial regions. Hence, more importance should be placed on this aspect.

Anderson et al. [15] indicated that patients were more prone to post-trauma arthritis when MCS exceeded 4.5 MPa in long-term follow-up. We obtained an associated IAIF defect size of 17.8 mm^2^ in the posterolateral region and 40.9 mm^2^ in the posteromedial region when the MCS exceeded 4.5 MPa. Therefore, IAIF defects greater than 17.8 mm^2^ in the posterolateral region and more than 40.9 mm^2^ in the posteromedial region should be reduced anatomically to reduce the occurrence of long-term post-trauma arthritis. The results are consistent with our other study showing that patients will suffer a poor prognosis and post-traumatic osteoarthritis if AIAIF is over 40 mm^2^ based on a large patient cohort [22]. Otherwise, the ankle joint will bear a maximum stress of 5 times the body weight when we run or jump in some activities. Therefore, for any size of defect, the impact on joint stress and stability should be considered in the treatment of ankle fractures to reduce damage caused by the IAIF defect.

McKinley et al. [14] found that contact stress peaks did not occur at the stepoff edge but rather occurred 2–3 mm away from the edge of the stepoff. Huber-Betzer et al. [23] attributed the phenomenon to the absence of buttressing at the lip of the step. In our study, the MCS was increased mainly in the border of the defect. We considered that there is a buttress around our IAIF defect. We divided the distal tibial articular surface into four quadrants: AOC, BOC, AOD and BOD. We recorded the MCS for each region and found that the MCS increases slowly with the increase in the size of the IAIF defect in the residual three regions, except for the region of the IAIF defect. The increased contact stress area also continuously increases with increasing IAIF defect size. There was a substantial impact on the distribution of MCS when the defect area was 42 mm^2^ in the posterolateral region. The MCS has a rapid increase in the BOD and AOD regions. For IAIF defects of the same size, the MCS in the posterolateral region is larger than that in the posteromedial region. This indicates a higher impact of the contact stress of the ankle joint when the IAIF defect is in the posterolateral region. The posterolateral IAIF defect in ankle fracture should be taken more seriously.

There were several limitations in this study. The mechanical properties of the ankle joint are quite complicated. Although our model can simulate the anatomy of the ankle joint and surrounding structures more realistically and accurately, it still results in little difference between finite element analysis results and actual situations. However, there is no influence on the size of the IAIF defect. The next step in our research is to further verify the results of this experiment in cadaver samples or clinical patients.

## Conclusion

In summary, a small IAIF defect in the posterior malleolus will result in a high MCS, and the MCS in the posterolateral region is larger than the MCS in the posteromedial region when the size of the IAIF defect is the same. We obtain the regression equation of MCS and area of IAIF defect. It has been indicated that patients are more prone to post-trauma arthritis when the size of IAIF defect is about more than 17.8 mm^2^ in posterolateral region and about more than 40.9 mm^2^ in posteromedial region. The MCS is increased mainly in the border of the IAIF defect. The increased contact stress area is also continuously increased with the increase in the size of the IAIF defect. These findings suggest that doctors should pay attention to IAIF defects in posterior malleolar fractures, and it is also helpful to develop optimal treatment protocols.

## Data Availability

The datasets and materials are available from corresponding authors on reasonable request.

## Abbreviations

IAIF: Intra-articular impacted fragment
MCS: Maximum contact stress
FEA: Finite element analysis
PTOA: Post-traumatic osteoarthritis
